# Short-term real-world effectiveness of faricimab on macular edema due to retinal vein occlusion

**DOI:** 10.1186/s40942-025-00703-3

**Published:** 2025-07-15

**Authors:** Toshiaki Hirakata, Ai Toride, Kenta Ashikaga, Takanori Nakagawa, Fumihiro Hara, Yuta Nochi, Shutaro Yamamoto, Yoshimune Hiratsuka, Shintaro Nakao

**Affiliations:** Department of Ophthalmology, Juntendo University Faculty of Medicine, Hongo 3-1-3, Bunkyo-ku, Tokyo, 113-8431 Japan

**Keywords:** PRN, Retinal vein occlusion, Faricimab, Macular edema, Vascular endothelial growth factor

## Abstract

**Background:**

Faricimab, the new anti-vascular endothelial growth factor (VEGF) drug including a bispecific antibody targeting both VEGF-A and angiopoietin-2 (Ang-2), has emerged as a therapeutic option for macular edema secondary to retinal vein occlusion (RVO), and its efficacy has been demonstrated in randomized controlled trials (RCTs); however, reports on its use in clinical practice are still limited. This study was conducted to evaluate the real-world treatment outcomes of faricimab for macular edema secondary to RVO, managed with a single initial injection plus pro re nata (1 + PRN) approach in both treatment-naïve and previously treated patients who switched to this regimen.

**Methods:**

This retrospective observational study included patients diagnosed with branch or central RVO, who received intravitreal faricimab therapy following the 1 + PRN protocol. Best-corrected visual acuity (BCVA) and central macular thickness (CMT) were analyzed.

**Results:**

Thirty patients (17 naïve and 13 switched) were included. The number of IVF was 1.4 ± 0.7 and 2.4 ± 2.1, in the naïve and switch groups, respectively. The mean follow-up period was 3.7 ± 2.7 and 4.9 ± 2.9 months in the naïve and switch patients, respectively. Mean LogMAR BCVA improved in the naïve group from 0.30 ± 0.37 at baseline to 0.11 ± 0.20 (*p* = 0.01) at the final visit, while there was no significant difference between 0.45 ± 0.45 at baseline and 0.35 ± 0.37 at the final visit in the switch group (*p* = 0.19). CMT reduction was significant in both groups; from 442 ± 117 μm at baseline to 304 ± 57 μm at one month after final IVF (*p* < 0.0001) in the naïve group; and from 436 ± 170 μm at baseline to 285 ± 76 μm at one month after final IVF (*p* = 0.0002) in the switch group.

**Conclusion:**

The 1 + PRN faricimab regimen improves vision and reduces macular edema with a reduced injection burden in patients with RVO. These findings validated the real-world efficacy of faricimab and supported its use as a viable therapeutic agent.

## Introduction

Retinal vein occlusion (RVO) is a leading cause of vision loss and is characterized by retinal ischemia and macular edema [1, 2]. Macular edema, which affects up to 15% of eyes with branch RVO (BRVO) and 30% of eyes with central RVO (CRVO), is the most frequent vision-threatening consequence of RVO [1, 2]. Intravitreal anti-vascular endothelial growth factor (anti-VEGF) therapy remains the primary treatment modality [3, 4]. Although fixed or treat-and-extend regimens have demonstrated efficacy, the 1 + PRN approach is increasingly utilized in clinical practice to balance efficacy and the treatment burden.

Faricimab, a bispecific antibody targeting both VEGF-A and angiopoietin-2 (Ang-2), has shown promising results in age-related macular degeneration [5] and diabetic macular edema [6, 7]. Subsequently, BALATON and COMINO trials, which are previously randomized controlled phase 3 trials (RCTs), have also proven the efficacy of faricimab for RVO [8]. In these RCTs, enrolled patients received intravitreal faricimab (IVF) 6.0 mg every 16 weeks, after six initial doses every four weeks, per a modified treat-and-extend regimen [9].

However, real-world data comparing naive and switched patients using this regimen are limited. This study evaluated the efficacy and treatment burden of the 1 + PRN strategy for RVO treated with faricimab in these two cohorts.

## Methods

This study included patients treated with IVF for macular edema of the RVO between March first, 2024 and December 31, 2024. The Inclusion criteria included patients aged 20 years or older with macular edema secondary to branch or central RVO, regardless of gender. Both ischemic and non-ischemic RVO cases were included. Some patients with ischemic RVO had previously undergone laser photocoagulation prior to the initiation of faricimab treatment. The exclusion criteria were cases that could not be followed up due to the complications of diseases that cause retinal exudative changes other than RVO during follow-up, and cases that self-interrupted their hospital visits. Patients with a history of vitrectomy were included. IVF was performed when retinal exudative changes (intraretinal and subretinal fluids) were detected using optical coherence tomography (OCT). In this study, all patients in the switch group had a history of three or more prior intravitreal anti-VEGF injections. Among them, patients were included if they met at least one of the following criteria: (1) persistent retinal fluid despite treatment, (2) inability to extend the treatment interval beyond 8 weeks, or (3) inability to discontinue injections despite more than two years of ongoing treatment. The administration method of IVF was performed using the 1 + PRN regimen. This study was conducted in accordance with the Declaration of Helsinki and approved by the Institutional Review Board of Juntendo University (16–292). Written informed consent from the patients enrolled in this study was waived, based on the opt-out method facilitated by our hospital bulletin board. All patient data were anonymized and retrospectively collected. The data were accessed and analyzed from March, 2024 to February 15, 2025.

The primary outcome of the study was a change in visual acuity from baseline. The secondary outcome was a change in central macular thickness (CMT) on OCT from baseline. All patients underwent comprehensive ophthalmic examinations, including a logarithmic examination of the minimum angle of resolution best-corrected visual acuity (logMAR BCVA); intraocular pressure (IOP); fundus ophthalmoscopy; and spectral-domain OCT. Changes in BCVA were assessed by comparing baseline values with those measured at the final visit during the follow-up period. CMT was assessed using SD-OCT. Two devices were used: Cirrus HD-OCT (Carl Zeiss Meditec, Dublin, CA, USA) with the 512 × 128 macular cube protocol, and Spectralis OCT (Heidelberg Engineering, Heidelberg, Germany) with the volume scan protocol. All measurements were performed consistently using either of these protocols depending on the device used. The same OCT measurement results were used for analysis for the same patients. Comparisons were made between baseline data, data one month after the last IVF administration, and data on the date of the last visit during the follow-up period. Statistical analyses were performed using the Wilcoxon matched-pairs signed rank test in Prism 10 (GraphPad Software). Normality was assessed using the Shapiro–Wilk test. As the data did not follow a normal distribution (*p* < 0.05), Statistical significance was set at *p* < 0.05. To confirm the safety of faricimab, adverse events related to its use during the study period were also investigated. Endophthalmitis, retinal detachment, and systemic adverse events were checked in agreement with the BALATON and COMINO trials [8].

## Results

This study included 35 consecutive eyes of 35 patients (17 male and 18 female) treated with IVF for macular edema of the RVO between March, 2024 and December 31, 2024. Five patients (five eyes) were excluded because they interrupted their visits and could not be followed up. Finally, 30 eyes of 30 patients (16 male and 14 female) were included in this study. Table 1 shows the demographic patients included in this study. They were divided into naïve (17 eyes of 17 patients) and switched (13 eyes of 13 patients) groups, and their medical records were retrospectively reviewed. Of the 13 patients in the switch group, 11 had an inadequate response and 2 were ineffective, which was the reason for the switch from previous anti-VEGF drugs. Of the 11 cases with inadequate efficacy, two required continued injections even after more than two years of prior anti-VEGF treatment, three showed slight improvement but persistent fluid remained, and six experienced recurrence within eight weeks. Two ineffective cases showed no reduction in retinal. The mean number of previous anti-VEGF drugs, including aflibercept, ranibizumab, and bevacizumab, was 16.2 ± 12.3, and the average duration of previous anti-VEGF therapy was 44.8 ± 30.2 months. In the switch group, prior anti-VEGF treatments included aflibercept (*n* = 7), ranibizumab (*n* = 5), and bevacizumab (*n* = 1). The mean follow-up period was 3.7 ± 2.7 and 4.9 ± 2.9 months in the naïve and switch patients, respectively. The interval between the final visit and the last IVF is 2.5 ± 2.1 in naïve group, and 2.1 ± 1.0 in switch group.

**Table 1:** Demographic patients

	Naïve	Switch	
Caes (eyes)	17 (17)	13 (13)	
Gender men (female)	8 (9)	8 (5)	*p* = 0.68
Age (years)	69.0 ± 9.9	64.0 ± 6.7	*p* = 0.001*
RVO type (cases)			*p* = 0.19
-BRVO	13	7	
-hemi-CRVO	0	2	
-CRVO	4	4	
Type of macular edema (cases)			*p* = 0.41
-IRF	16	10	
-IRF + SRF	1	3	
The number of IVF	1.4 ± 0.8	2.4 ± 2.1	*p* = 0.14
Mean follow-up period (month)	3.7 ± 2.7	4.9 ± 2.9	*p* = 0.30
The number of previous anti-VEGF drug		16.2 ± 12.3	
Past history of laser photocoagulation (cases)	2	3	
Past history of vitrectomy (cases)	0	1	
ERM before IVF (cases)	2	2	
New ERM after IVF (cases)	0	0	

BRVO, branch retinal vein occlusion; CRVO, central retinal vein occulusion; ERM, epiretinal membrane; IRF, inner retinal fluid; IVF, intravitreal faricimab RVO, retinal vein occlusion; SRF, subretinal fluid

Statistical analysis was analyzed by the chi-square test or an unpaired two-sample *t*-test. A *p*-value of less than 0.05 was considered statistically significant

In the naïve group, 13 patients had BRVO and 4 patients had CRVO. In the switched group, 7 patients had BRVO, 2 had hemi-CRVO, and 4 had CRVO. Focal laser photocoagulation of the non-perfusion area was performed in 2 patients (both BRVO) in the naïve group and 3 patients (1 BRVO, 1 CRVO, and 1 hemi-CRVO) in the switch group, prior to the initiation of faricimab treatment. Two patients each in the naïve group and two cases of the switch group had epiretinal membrane (ERM) on OCT at baseline. One eye from one patient in the switch group, who had a history of vitrectomy for rhegmatogenous retinal detachment retinal detachment, was included in this study. The number of IVF was 1.4 ± 0.7 and 2.4 ± 2.1, in both the naïve and switch groups respectively.

**Fig. 1:**
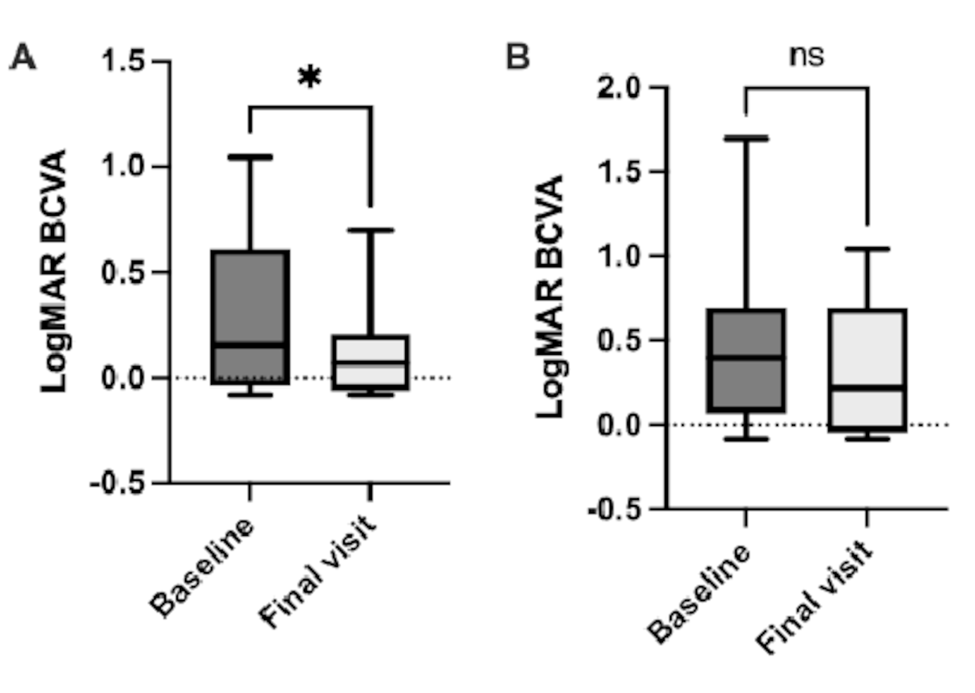
Mean LogMAR best-corrected visual acuity (BCVA) changes by treatment with faricimab. (**A**) In the naïve group, the mean logMAR BCVA improved significantly between baseline and final visit. (**B**) In the switched group, the mean LogMAR BCVA did not improve significantly between the baseline and final visit. Error bars represent standard deviation. *P*-values are indicated by asterisks (* *P* < 0.05)

LogMAR improved significantly from 0.30 ± 0.37 at baseline to 0.11 ± 0.20 at the final visit during the follow-up period (*p* = 0.01) in the naïve group (Fig. 1A). On the other hand, in the switch group, LogMAR BCVA was not significantly different between 0.45 ± 0.45 at baseline and 0.35 ± 0.37 at the final visit (*p* = 0.19, Fig. 1B).

Morphological improvement was observed in both the naïve and switch groups. One month after the initial IVF, 12 of 17 (71%) eyes in the naïve group and 6 of 13 (46%) eyes in the switch group showed a disappearance of macular retinal fluid on OCT. Moreover, 13 of 17 (76%) eyes in the naïve group and 12 of 13 (92%) eyes in the switch group achieved CMT under 325 μm at one month after the initial IVF. At one month after the final intravitreal faricimab injection, 12 of 17 patients (70.6%) in the naïve group achieved CMT under 300 μm, and 2 of 17 patients (11.8%) achieved CMT under 250 μm. In the switch group, 9 of 13 patients (69.2%) achieved a CMT of less than 300 μm, and 6 of 13 patients (46.2%) achieved a CMT of less than 250 μm.

**Fig. 2:**
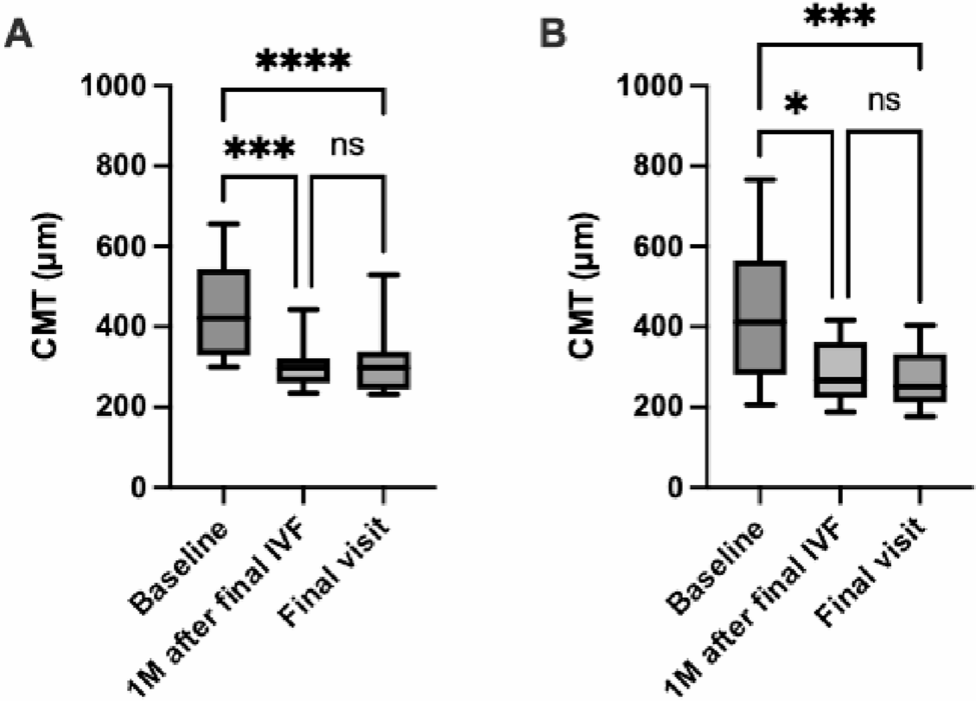
Mean central macular thickness (CMT) changes by treatment with faricimab. (**A**) In the naïve group, CMT was significantly reduced at both one month after the final intravitreal faricimab (IVF) and at the final visit, compared with baseline. (**B**) In the switched group, CMT was also significantly reduced at both one month after the final IVF, and at the final visit, compared with baseline. Error bars represent standard deviation. *P*-values are indicated by asterisks (* *p* < 0.05, ** *p* < 0.01, and **** *p* < 0.0001)

In the naïve group, CMT decreased from 442 ± 117 μm at baseline to 304 ± 56 μm at one month after final IVF (*p* = 0.0002); and the CMT improvement was maintained at the final visit (313 ± 78 μm, *p* < 0.0001) of the follow-up period (Fig. 2A). In the switch group, CMT decreased from 436 ± 170 μm at baseline to 285 ± 76 μm at one month after the final IVF (*p* < 0.05); and the CMT improvement was maintained at the final visit (271 ± 71 μm, *p* = 0.0002) of the follow-up period (Fig. 2B).

In this study, two patients in the naïve group and two patients in the switch group had ERM at baseline. When all four cases combined are analyzed for changes in CMT, CMT was 471 ± 110 μm, 340 ± 43 μm (*p* = 0.056), and 333 ± 48 μm (*p* = 0.051), at baseline, 1 month after final IVF, and final visit, respectively. During the follow-up period, no new ERM were observed in any of the patients on OCT; and no significant ocular or systemic adverse events were observed.

Both baseline IOP before IVF treatment (baseline) and the final IOP (final visit) were in the normal range and did not differ either in the naïve group or in the switched groups (14.2 ± 3.6 mmHg and 13.9 ± 2.7 mmHg (*p* = 0.65), and 13.7 ± 3.0 mmHg and 14.5 ± 3.6 mmHg (*p* = 0.37)).

## Discussion

To the best of our knowledge, this is the first report of real-world cohort to evaluate the efficacy of faricimab in RVO-related macular edema in Japan. In this study, we demonstrated the efficacy of the 1 + PRN faricimab regimen for the treatment of RVO. Visual acuity improved in the naïve group; however, no significant improvement was observed in the switch group. The CMT improved in both the naïve and switch groups.

The BALATON and COMINO trials reported good long-term results, in which visual acuity gains and CST reductions achieved at week 24 were maintained through week 72 [10]. In these RCTs, patients randomized to faricimab 6.0 mg every four weeks (Q4W) up to week 20 received faricimab (6.0 mg), and were dosed per a modified treat and extend (TAE)-based regimen from weeks 24–72, which treatment intervals were adjusted based solely on retinal fluid status as determined by OCT findings and the interval was extended or shortened in 2-week increments within a range of 4 to 16 weeks. On the other hand, another available real-world cohort study showed that patients with BRVO-related ME received a mean of 4.5, with a median of 5 anti-VEGF injections in six months, and a mean of 7.4 with a median of 8 injections in one year; patients with CRVO-related ME received a mean of 4.6 with a median of 5 anti-VEGF injections in six months, and a mean of 7.6 anti-VEGF injections with a median of 8 injections in one year [11]. These reports suggest that the number of injections required for RVO in actual clinical practice may be fewer than in RCTs. Indeed, in our study, the number of IVF was 1.4 ± 0.7 /3.7 ± 2.7 months and 2.4 ± 2.1 / 4.9 ± 2.9 months in both the naïve and switch groups respectively. However, the lower number of injections observed in this study compared to RCTs may reflect under-treatment, which is common in real-world settings due to patient and clinic-related factors. Therefore, these findings should be interpreted with caution, and longer-term studies and prospective studies are needed for verification.

Real-world data on faricimab for RVO remain scarce. To our knowledge, the only published real-world study to date is the retrospective analysis by Arslan et al., which investigated the short-term response to faricimab during the loading phase in 19 eyes with RVO [12]. The patients had been previously treated with other anti-VEGF agents and were switched to faricimab, receiving four consecutive monthly injections. During 3-month observation period, patients showed notable improvements in visual acuity and retinal morphology. On the other hand, a recent meta-analysis by Chung et al. compared the efficacy of a 3-monthly loading injection followed by a PRN regimen (3 + PRN) with a single injection followed by PRN (1 + PRN) in patients with BRVO, using ranibizumab, aflibercept, or bevacizumab [13]. Across six studies, including one RCT, there were no significant differences between the two regimens in terms of visual or anatomical outcomes. However, the total number of injections was significantly lower in the 1 + PRN group, suggesting a reduced treatment burden without compromising efficacy. Our study is one of the first to report real-world outcomes of a 1 + PRN regimen with faricimab in both naïve and previously treated eyes. The importance of the loading phase in faricimab will be verified in the future through long-term prognosis and prospective studies.

Given this limited evidence base, it is helpful to contextualize our findings by comparing them with other real-world studies employing different anti-VEGF regimens, such as fixed dosing or TAE strategies. For instance, Rodríguez‑Fernández et al. reported favorable outcomes in BRVO using a TAE approach with ranibizumab, achieving both visual improvement and reduced injection burden over two years [tr^14^]. Ciulla et al. analyzed over 15,000 eyes with macular edema due to RVO and found that while real-world injection frequencies were lower than in clinical trials, visual outcomes were directly correlated with the number of injections administered [11]. These findings underscore the importance of consistent follow-up and timely reinjection, even beyond the initial intensive treatment period. Compared to these studies, our 1 + PRN approach demonstrated that reasonable anatomical and functional outcomes can be achieved with fewer injections during short-term follow-up, particularly when disease activity is monitored and treated in a timely manner. However, the potential advantages of TAE regimens—including proactive extension of treatment intervals and reduced recurrence—merit further investigation in the context of faricimab therapy.

In the switch group, no improvement in visual acuity was observed as a therapeutic effect; however, improvements in retinal morphology that had not been achieved with conventional medications were observed. A higher expression of Ang-2 contributes to the pathophysiology of RVO [15]. Faricimab possesses anti-Ang-2 activity, which was absent in previous anti-VEGF agents. Anti-Ang-2 activity contributes to vascular stability, and therefore, it may hold promise for the treatment of RVO.

No significant difference in visual acuity was observed in the switch group; however, a tendency toward improvement was observed. It suggests that anatomical improvements may be effective in improving or maintaining long-term vision in switched cases. On the other hand, similar observations have been reported in real-world studies of faricimab for diabetic macular edema [16, 17]. In these studies, evaluating patients switched to faricimab after previous anti-VEGF therapy, significant anatomical improvements were observed—particularly reductions in central subfield thickness—while visual acuity remained stable without significant gains. It was suggested that chronic retinal damage might limit functional recovery even when morphological outcomes improve. Therefore, a longer follow-up period is required.

Interestingly, two patients in the naïve group and two patients in the switch group had ERM at baseline, indicating a reduction in CMT. It suggests that faricimab is effective even in patients with ERM. During the follow-up period, no new ERMs were detected. In addition, no ocular complications such as intraocular inflammation, retinal vascular occlusion, or uveitis, or systemic complications such as cerebrovascular or cardiac disease were observed during the observation period of this study.

Our study has several limitations, including its retrospective design, relatively small sample size, and short follow-up period. The difference in follow-up duration between the naïve and switch groups, along with the variability, represents a limitation in the direct comparison of injection frequency. Therefore, future studies should include prospective, large-scale trials with longer observation periods to validate these findings. Direct comparisons with other anti-VEGF agents under different treatment regimens (e.g., fixed dosing, treat-and-extend) are also warranted to determine the relative efficacy and durability of faricimab in real-world settings. Additionally, identifying predictive markers of treatment response may support individualized therapeutic strategies.

This study investigated short-term treatment outcomes of faricimab for RVO in a clinical setting. All patients were treated with a 1 + PRN regimen, and improvements in retinal thickness were observed in both the naive and switch groups. Additionally, improvements in visual acuity were noted in the naive group. These findings suggest that faricimab may be effective for RVO with fewer injections required than in RCTs.

## Data Availability

No datasets were generated or analysed during the current study.

## Abbreviations

Ang-2: Angiopoietin-2
BCVA: Best-corrected visual acuity
BRVO: Branch retinal vein occlusion
CMT: Central macular thickness
CRVO: Central retinal vein occlusion
IVF: Intravitreal faricimab
LogMAR: Logarithmic examination of the minimum angle
OCT: Optic coherence tomography
PRN: Pro re nata
RCT: Randomized controlled trials
RVO: Retinal vein occlusion
TAE: Treat and extend
VEGF: Vascular endothelial growth factor
